# Increasing insecticide resistance in *Anopheles funestus* and *Anopheles arabiensis* in Malawi, 2011–2015

**DOI:** 10.1186/s12936-016-1610-1

**Published:** 2016-11-22

**Authors:** Themba Mzilahowa, Martin Chiumia, Rex B. Mbewe, Veronica T. Uzalili, Madalitso Luka-Banda, Anna Kutengule, Don P. Mathanga, Doreen Ali, John Chiphwanya, John Zoya, Shadreck Mulenga, Wilfred Dodoli, Jennifer Bergeson-Lockwood, Peter Troell, Jessica Oyugi, Kim Lindblade, John E. Gimnig

**Affiliations:** 1 Malaria Alert Centre, Malawi College of Medicine, Chichiri, P/Bag 360, Blantyre 3, Malawi; 2 Community Health Services Unit, National Malaria Control Programme, Ministry of Health, Lilongwe, Malawi; 3 WHO Country-office, Lilongwe, Malawi; 4 President’s Malaria Initiative, United States Agency for International Development, Lilongwe, Malawi; 5 President’s Malaria Initiative, Centers for Disease Control and Prevention, Lilongwe, Malawi; 6 Division of Parasitic Diseases and Malaria, Centers for Diseases Control and Prevention, Atlanta, GA USA

**Keywords:** *Anopheles funestus*, *Anopheles gambiae*, Insecticide resistance, Pyrethroid resistance, Malawi

## Abstract

**Background:**

Susceptibility of principal *Anopheles* malaria vectors to common insecticides was monitored over a 5-year period across Malawi to inform and guide the national malaria control programme.

**Methods:**

Adult blood-fed *Anopheles* spp. and larvae were collected from multiple sites in sixteen districts across the country between 2011 and 2015. First generation (F_1_) progeny aged 2–5 days old were tested for susceptibility, using standard WHO procedures, against pyrethroids (permethrin and deltamethrin), carbamates (bendiocarb and propoxur), organophosphates (malathion and pirimiphos-methyl) and an organochlorine (DDT).

**Results:**

Mortality of *Anopheles funestus* to deltamethrin, permethrin, bendiocarb and propoxur declined significantly over the 5-year (2011–2015) monitoring period. There was wide variation in susceptibility to DDT but it was not associated with time. In contrast, *An. funestus* exhibited 100% mortality to the organophosphates (malathion and pirimiphos-methyl) at all sites tested. There was reduced mortality of *Anopheles arabiensis* to deltamethrin over time though this was not statistically significant. However, mortality of *An. arabiensis* exposed to permethrin declined significantly over time. *Anopheles arabiensis* exposed to DDT were more likely to be killed if there was high ITN coverage in the mosquito collection area the previous year. There were no other associations between mosquito mortality in a bioassay and ITN coverage or IRS implementation. Mortality of *An. funestus* from four sites exposed to deltamethrin alone ranged from 2 to 31% and from 41 to 94% when pre-exposed to the synergist piperonyl butoxide followed by deltamethrin. For permethrin alone, mortality ranged from 2 to 13% while mortality ranged from 63 to 100% when pre-exposed to PBO.

**Conclusion:**

Pyrethroid resistance was detected in *An. funestus* and *An. arabiensis* populations across Malawi and has worsened over the last 5 years. New insecticides and control strategies are urgently needed to reduce the burden of malaria in Malawi.

**Electronic supplementary material:**

The online version of this article (doi:10.1186/s12936-016-1610-1) contains supplementary material, which is available to authorized users.

## Background

Malaria control in sub-Saharan Africa includes the distribution of long-lasting insecticidal nets (LLINs), indoor residual spraying of houses, intermittent preventive treatment of pregnant women and prompt diagnosis and treatment of clinical malaria. The scale-up of these interventions has resulted in a reduction in the burden of malaria, particularly in sub-Saharan Africa. According to recent World Malaria Reports [1, 2], a significant reduction in malaria incidence (37%) and mortality (48%) has been reported since 2000. Most of the gain in the reported malaria reduction has been attributed to vector control, mainly widespread use of LLINs [3]. Despite the general reduction in malaria regionally, disease burden remains high in Malawi [4–8] with approximately 4 million new malaria cases reported at out-patient departments in hospitals and health facilities across the country in 2015 and an additional 2.1 million cases reported by community based health workers [9].

Insecticide resistance poses a major challenge to malaria vector control and thus threatens malaria control efforts in sub-Saharan Africa. Pyrethroid resistance was first reported in *Anopheles gambiae* in West Africa where it was attributed to widespread use of insecticides in agriculture [10]. Resistance in *An. gambiae* has since been reported from nearly all countries in sub-Saharan Africa [11]. The geographical extent or status of the problem has recently been well documented [12–14]. Though evidence for its impact on the effectiveness of LLINs is limited [15, 16], pyrethroid resistance has been associated with an epidemic of malaria in South Africa [17] and has necessitated a change in insecticides for IRS in nearly all countries in sub-Saharan Africa where IRS programs were active. The increased costs associated with non-pyrethroids has resulted in reduced geographic coverage of IRS and in some cases, has resulted in the abandonment of IRS programmes [18].

Given the potential impact of insecticide resistance on malaria control programmes, it is essential to monitor mosquito populations to help guide the implementation of vector control interventions. Insecticide resistance status and its geographical extent in *Anopheles* vector populations has been monitored in Malawi since 2007. Results of the observations carried out between 2007 and 2011 were published elsewhere [19]. In this paper, we present results of resistance situation across Malawi between 2011 and 2015 within populations of the two principal malaria vector populations, *An. funestus* sensu stricto and *An. gambiae* sensu lato.

## Methods

### Study sites

Data were collected from 16 of the 28 districts in Malawi between 2011 and 2015 (Additional file 1: Table S1). Use of insecticide-treated nets (ITNs) for malaria control has been national policy in Malawi since 2002 using a two-pronged approach. First, a net is given to a pregnant woman at her first antenatal visit to the clinic and a second net is given after the birth of the new-born baby. Second, LLINs are distributed to the general population through mass net campaigns approximately every three years with one conducted during the study period in 2012 and the follow up due in 2015/16 calendar year. Further, between 2007 and 2013, six of the above districts (Mangochi, Chikwawa, Salima, Nkhotakota, Karonga and Nkhata Bay) received at least two annual rounds of IRS funded by either the Government of Malawi or the President’s Malaria Initiative (PMI) (Table 1).

**Table 1:** Six districts that have had two or more rounds of indoor residual spraying (IRS)

District	IRS rounds	Year(s) sprayed	Insecticide sprayed
Mangochi	2	2010, 2012	Alphacypermethrin
Chikwawa	2	2010, 2012	Alphacypermethrin
Salima	2	2010, 2012, 2013	Lambdacyhalothrin; pirimiphos-methyl
Nkhotakota	5	2007–2011	Alphacypermethrin; lambdacyhalothrin; pirimiphos-methyl
Nkhata Bay	2	2010, 2012	Alphacypermethrin
Karonga	2	2010, 2012	Alphacypermethrin

### Mosquito collection

Battery operated aspirators [20] were used to collect live, blood-fed, female *Anopheles* mosquitoes resting inside houses. In almost all study districts, collections were normally carried out at two sites or villages in each district. Study sites were selected based on district malaria prevalence data with highly endemic catchment areas being prioritized. Study sites within each district varied by year to find areas with large numbers of mosquitoes, with the aim of 100 mosquitoes for each site and insecticide. At each site, as many houses were sampled as needed to collect adequate numbers of blood fed *Anopheles* mosquitoes. During later years including 2015, collections of mosquito larvae were carried out to supplement indoor collections and to ensure a representative sampling of *An. gambiae* s.l. All samples were transported to the insectaries at the Malaria Alert Centre (MAC) of the Malawi College of Medicine in Blantyre for rearing to obtain first generation (F_1_) adults.

For mosquitoes collected as blood-fed or gravid adults, eggs were obtained from individual females through a forced egg-laying technique developed by the Vector Research Group of the Liverpool School of Tropical Medicine (LSTM) and described by the Malaria Research and Reference Reagent Resource Center (MR4) Egg batches from individual females were placed in separate plastic cups containing mineral water. After hatching, larvae were fed on finely ground fish food pellets (TOPFIN; Pond Fish Food; Pacific Coast Distributing Inc., USA) until second instar stage when they could confidently be identified as belonging either to *An. gambiae* s.l. or *An. funestus* group of mosquitoes. Larvae were then pooled by species and transferred into larger larval trays. Mineral water was changed every 3 days until they started pupating. All pupae were picked and placed in plastic cages (BugDorm-1 Insect Rearing Cage w/Screen Port measuring 30 × 30 × 30 cm; MegaView Science Co., Ltd) by species. The emergent adults, aged 2–5 days old were then tested against the various insecticides. Mosquitoes collected as larvae were reared in mineral water and fed on fish food as described above until adults emerged and were subsequently tested.

### Susceptibility testing

The WHO test kits and insecticide-treated papers were used to test mosquitoes for resistance. First generation (F_1_) progeny aged 2–5 days old were tested throughout the experiments following standard WHO test procedures [21]. Briefly, for each insecticide tested, 20–25 female *Anopheles* mosquitoes were exposed for 60 min in a single tube and four replicates were performed. After exposure, mosquitoes were transferred into resting tubes and provided with 10% sugar solution. Mortality was assessed 24 h post exposure. For each insecticide tested, a corresponding control assay was carried out in which 20–25 mosquitoes were tested with untreated papers. Positive controls were run using a colony of susceptible *An. gambiae* Kisumu strain to test the quality of the insecticide-treated papers. Insecticides for testing were prioritized in descending order as follows: pyrethroids > carbamates > organophates > organochlorines. This was done for programmatic purposes as pyrethroids are most commonly used in public health.

In order to deduce mechanism of resistance to pyrethroids, we pre-exposed test mosquito samples to a synergist, piperonyl butoxide (PBO) using standard CDC bottle assays procedures. Firstly, Wheaton bottles were coated with PBO at a concentration of 400 µg/bottle dissolved in acetone as a solvent. Females were first exposed to PBO for 1 h followed by exposure to a test pyrethroid for another 1 h.

A sub-sample (>80%) of both resistant and susceptible specimens were subsequently subjected to PCR in order to identify individual sibling species within *An. funestus* group of mosquitoes and *An. gambiae* species complex [22, 23].

### Data analysis

Data from the same location (district and village) and date were combined for each species across all bioassays. If control mortality was >10%, the data were excluded from analysis. Abbott’s correction was not applied to bioassays where control mortality was between 5 and 10% [21], as this would have precluded some of the subsequent analyses. The proportion that was killed in each bioassay, along with 95% confidence limits, were calculated using the SURVEYFREQ procedure in SAS (SAS Institute, Inc., Cary, NC, USA). Estimates of 95% confidence limits were adjusted for correlation of mosquitoes tested within the same WHO assay tube. Since the SURVEYFREQ procedure could not estimate confidence limits in cases where 0 or 100% of mosquitoes died, confidence limits in these cases were manually estimated using the online calculator for proportions in OpenEpi and taking the Fisher Exact (Clopper-Pearson) estimates for the 95% confidence limits. Trends over time were first estimated by considering aggregate mortalities at each site and time and performing a regression of mortality versus the Julian dates of bioassays using the GLM procedure in SAS. Since linear regression models of proportions may be affected by the sample size used to estimate each point, logistic regression was used to estimate the association between year, IRS the previous year and ITN coverage the previous year. Year was converted to 0 through 4 and entered into the model as a continuous categorical variable. IRS was included as a continuous variable for the cumulative number of rounds of IRS with a pyrethroid insecticide for each district, as reported by the National Malaria Control Programme. Spraying in Salima and Nkhotakota districts with pirimiphos-methyl in late 2010 and late 2011 (Nkhotakota only) was accounted for by considering those years to be negative in the cumulative number of rounds of IRS. ITN coverage was obtained from from Malaria Indicator Surveys conducted in 2010, 2012 and 2014. For intervening years where data was unavailable, ITN coverage for each district was interpolated from existing data. ITN coverage was then converted into high coverage districts (≥70% net ownership) and medium coverage districts (<70% coverage). Separate models were generated for each species and insecticide. Data for *An. funestus* exposed to pirimiphos-methyl were combined with that of malathion as data were only available from one site and year. All models were generated in the GENMOD procedure in SAS and all models included the tube in which mosquitoes were tested as the repeated subject.

## Results

**Fig. 1:**
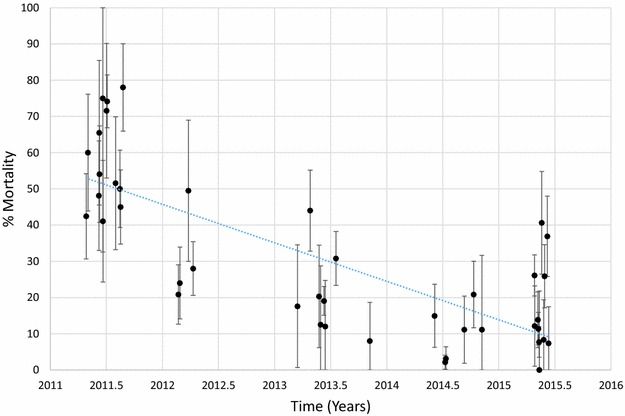
Mortality of *An. funestus* exposed to deltamethrin over time. Each *point* denotes the mortality for a single population with 95% confidence limits. The *X-axis* represents time expressed as the year plus the day of the year divided by 365.25

**Fig. 2:**
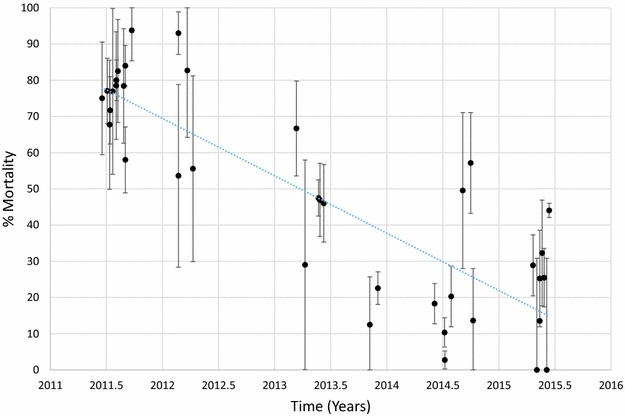
Mortality of *An. funestus* exposed to permethrin over time. Each *point* denotes the mortality for a single population with 95% confidence limits. The *X-axis* represents time expressed as the year plus the day of the year divided by 365.25

**Fig. 3:**
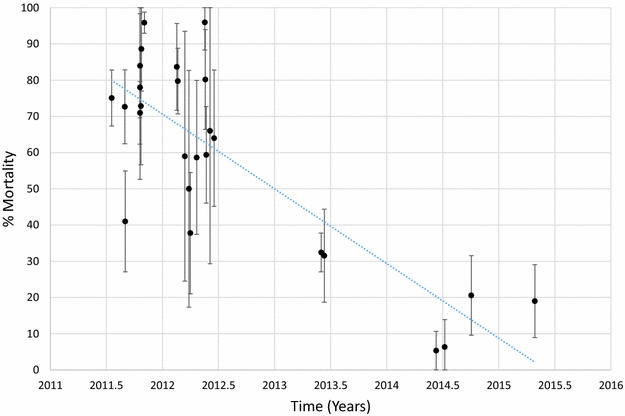
Mortality of *An. funestus* exposed to bendiocarb over time. Each *point* denotes the mortality for a single population with 95% confidence limits. The *X-axis* represents time expressed as the year plus the day of the year divided by 365.25

**Fig. 4:**
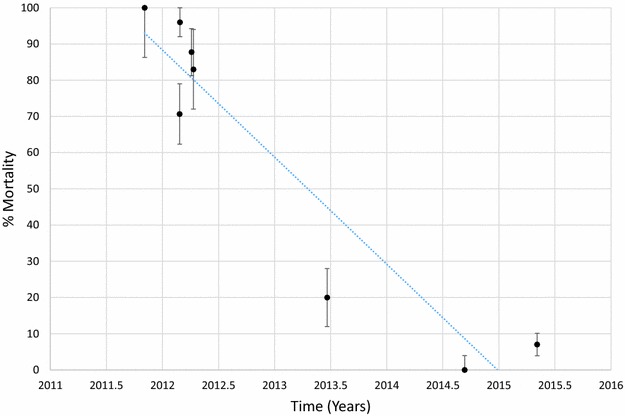
Mortality of *An. funestus* exposed to propoxur over time. Each *point* denotes the mortality for a single population with 95% confidence limits. The *X-axis* represents time expressed as the year plus the day of the year divided by 365.25

**Fig. 5:**
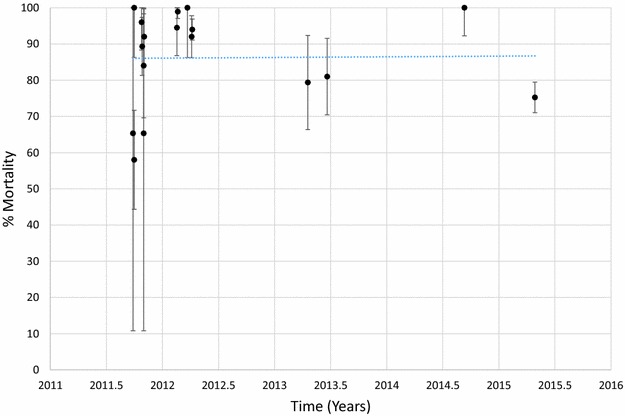
Mortality of *An. funestus* exposed to DDT over time. Each *point* denotes the mortality for a single population with 95% confidence limits. The *X*-*axis* represents time expressed as the year plus the day of the year divided by 365.25

**Fig. 6:**
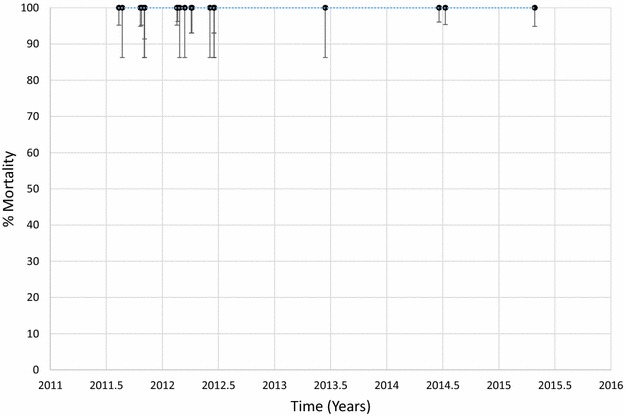
Mortality of *An. funestus* exposed to malathion over time. Each *point* denotes the mortality for a single population with 95% confidence limits. The *X-axis* represents time expressed as the year plus the day of the year divided by 365.25

Mortality of *An. funestus* to deltamethrin is presented graphically in Fig. 1 and provided in Additional file 1: Table S2. Morality to deltamethrin ranged from 45 to 78% in 2011 but declined over time with mortalities ranging from 0 to 41% in 2015. Considering each site and time as a single data point and estimating the trend over time, there was a significant negative correlation between mortality and time (p < 0.001). Similar results were obtained for *An. funestus* tested against permethrin (Fig. 2; Additional file 1: Table S3), bendiocarb (Fig. 3; Additional file 1: Table S4) and propoxur (Fig. 4; Additional file 1: Table S5). For permethrin, mortality ranged between 58 and 94% in 2011 but fell to 0–44% in 2015 with a significant negative trend over time (p < 0.001). For bendiocarb, mortalities were between 41 and 96% in 2011. There were fewer sites tested in 2013, 2014 and 2015 but mortality was 32% at two sites in 2013, 5, 6 and 21% at 3 sites in 2014 and 19% at one site in 2015. Again, there was a significant negative trend with mortality declining over time (p < 0.001). Mortality to propoxur was 100% in 2011 at a single study site and ranged between 71 and 96% at 4 sites in 2012. Mortality was 20% at one site in 2013 and was 0 and 7% at a different site in 2014 and 2015. The trend for declining mortality against propoxur was statistically significant (p < 0.001). Although there was considerable variation among sites and years in the morality of *An. funestus* exposed to DDT (Fig. 5; Additional file 1: Table S6), there was no clear trend over time (p = 0.953). Lastly, *An. funestus* exhibited 100% mortality to the organophosphate insecticides malathion and pirimiphos-methyl at all sites tested (Fig. 6; Additional file 1: Table S7). Maps showing the distribution of resistance in *An. funestus* over time are provided in Additional file 2: Figures S1–S3.

**Fig. 7:**
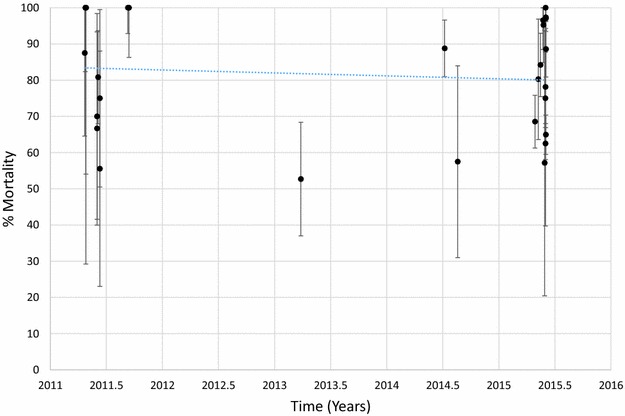
Mortality of *An. arabiensis* exposed to deltamethrin over time. Each *point* denotes the mortality for a single population with 95% confidence limits. The *X-axis* represents time expressed as the year plus the day of the year divided by 365.25

**Fig. 8:**
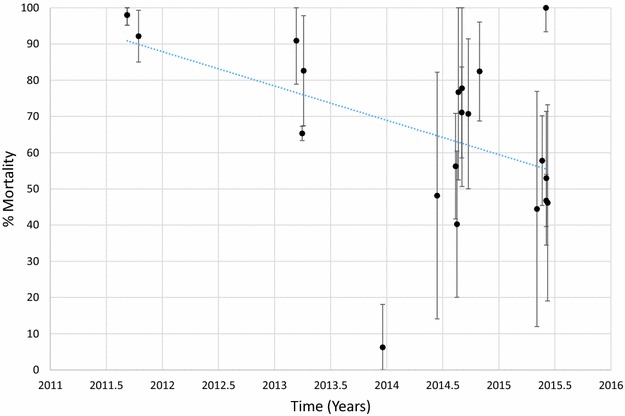
Mortality of *An. arabiensis* exposed to permethrin over time. Each *point* denotes the mortality for a single population with 95% confidence limits. The *X-axis* represents time expressed as the year plus the day of the year divided by 365.25

**Fig. 9:**
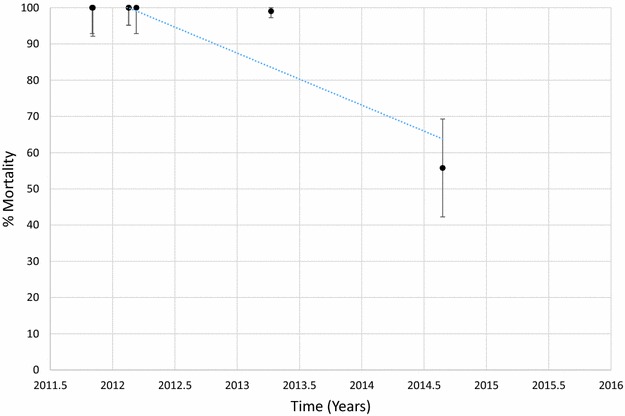
Mortality of *An. arabiensis* exposed to bendiocarb over time. Each *point* denotes the mortality for a single population with 95% confidence limits. The *X-axis* represents time expressed as the year plus the day of the year divided by 365.25

Mortality of *An. arabiensis* to deltamethrin is shown in Fig 7 and Additional file 1: Table S8. Mortality ranged between 56 and 100% in 2011 and between 57 and 100% in 2015. There was no evidence for a trend that was significantly different from zero (p = 0.626). In contrast, there was a slight decline in mortality of *An. arabiensis* exposed to permethrin over time (Fig. 8; Additional file 1: Table S9, p = 0.021). Mortality in this species was >90% at 3 sites tested in 2011 but was <60% in all sites tested in 2015 except for one in Mwanza district where mortality was 100% (Fig. 9; Additional file 1: Table S10). There were few sites where *An. arabiensis* was exposed to bendiocarb but a general declining trend was observed (p = 0.007). Maps showing the distribution of resistance in *An. arabiensis* over time are provided in Additional file 2: Figures S4–S6.

**Fig. 10:**
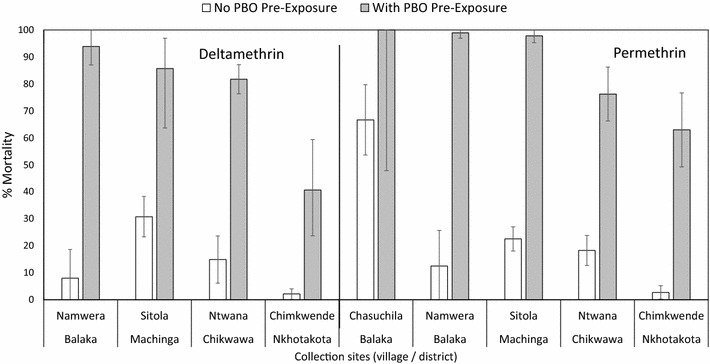
Mortality of different populations of *An. funestus* exposed to deltamethrin (*left side*) or permethrin (*right side*). *Open bars* indicate mortality of *An. funestus* without PBO pre-exposure while the *solid bars* indicate mortality after pre-exposure to PBO

Additional bioassays using *An. funestus* were done in 2013 and 2014 using a pre-exposure to piperonyl-butoxide, a synergist that interferes with the activity of oxidase enzymes that are involved in pyrethroid resistance. Paired data were available from four sites for deltamethrin and five sites for permethrin. At the four sites where deltamethrin was tested without PBO pre-exposure, mortality ranged from 2 to 31% mortality. At the same sites, mortality to deltamethrin after exposure to PBO ranged from 41 to 94%. For permethrin alone, mortality in mosquitoes ranged from 2 to 23%. After exposure to PBO, mortality among *An. funestus* exposed to permethrin ranged from 63 to 100% (Fig. 10).

A summary of logistic regression models exploring the effects of year, cumulative rounds of IRS and ITN coverage the previous year are presented in Table 2 for *An. funestus* and Table 3 for *An. arabiensis*. More detailed information is provided in Additional file 1: Tables S2–S8. For *An. funestus*, there was a significant decline in mortality over time for mosquitoes exposed to deltamethrin, permethrin, bendiocarb and propoxur. On average, the odds of mortality in *An. funestus* declined by 45.1% for deltamethrin, 45.9% for permethrin, 59.0% for bendiocarb and 89.8% for propoxur. Odds of mortality for *An. arabiensis* exposed to deltamethrin was not significantly associated with year while the odds of mortality in *An. arabiensis* exposed to permethrin declined by 44.3% each year. The odds of mortality of *An. funestus* exposed to DDT was significantly lower for areas with low ITN coverage (OR = 0.163, p = 0.014). There were no other associations between ITN coverage the previous year and the odds of mortality in a bioassay. There were no associations between the odds of mortality and cumulative rounds of IRS for any species or insecticide.

**Table 2:** Model table for *An. funestus* mortality in WHO susceptibility tests when exposed to deltamethrin, permethrin, bendiocarb, propoxur or DDT

Parameter	Level	Risk ratio	Lower CL	Upper CL	Z	Prob (Z)
*Deltamethrin*						
Intercept		1.058	0.817	1.369	0.426	0.670
Year		0.549	0.458	0.657	−6.538	<0.001
Cumulative IRS		1.380	0.994	1.917	1.925	0.054
Net ownership	1 = High	1.272	0.737	2.195	0.864	0.388
Previous year	2 = Low	Ref.	Ref.	Ref.		
*Permethrin*						
Intercept		3.267	2.456	4.347	8.127	<0.001
Year		0.541	0.466	0.628	−8.086	<0.001
Cumulative IRS		1.122	0.836	1.506	0.769	0.442
Net ownership	1 = High	0.754	0.487	1.169	−1.261	0.207
Previous year	2 = Low	Ref.	Ref.	Ref.		
*Bendiocarb*						
Intercept		2.429	1.346	4.382	2.946	0.003
Year		0.410	0.327	0.514	−7.736	<0.001
Cumulative IRS		1.202	0.929	1.555	1.396	0.163
Net ownership	1 = High	1.680	0.862	3.275	1.524	0.128
Previous year	2 = Low	Ref.	Ref.	Ref.		
*Propoxur*						
Intercept		51.025	12.451	209.101	5.464	<0.001
Year		0.102	0.042	0.245	−5.106	<0.001
Cumulative IRS		0.982	0.299	3.225	−0.030	0.976
Net ownership	1 = High	0.509	0.088	2.955	−0.753	0.452
Previous year	2 = Low	Ref.	Ref.	Ref.		
*DDT*						
Intercept		23.061	6.447	82.487	4.826	<0.001
Year		0.885	0.618	1.265	−0.671	0.502
Cumulative IRS		0.601	0.219	1.652	−0.987	0.324
Net ownership	1 = High	0.163	0.039	0.692	−2.461	0.014
Previous year	2 = Low	Ref.	Ref.	Ref.		

The probability that an individual mosquito died was modeled in a logistic regression using generalized estimating equations and adjusting for repeated measures within the same exposure tube

**Table 3:** Model table for *An. arabiensis* mortality in WHO susceptibility tests when exposed to deltamethrin or permethrin

Parameter	Level	Risk ratio	Lower CL	Upper CL	Z	Prob (Z)
*Deltamethrin*						
Intercept		3.344	1.909	5.860	4.219	<0.001
Year		0.875	0.753	1.017	−1.738	0.082
Cumulative IRS		1.106	0.805	1.519	0.623	0.533
Net ownership	1 = High	1.829	1.155	2.897	2.575	0.010
Previous year	2 = Low	Ref.	Ref.	Ref.		
*Permethrin*						
Intercept		8.902	4.088	19.387	5.506	<0.001
Year		0.557	0.459	0.676	−5.929	<0.001
Cumulative IRS		0.954	0.713	1.275	−0.320	0.749
Net ownership	1 = High	1.494	0.873	2.557	1.463	0.143
Previous year	2 = Low	Ref.	Ref.	Ref.		

The probability that an individual mosquito died was modeled in a logistic regression using generalized estimating equations and adjusting for repeated measures within the same exposure tube

## Discussion

This study presents comprehensive longitudinal data on the status of insecticide resistance in *An. funestus* and *An. arabiensis* to all four classes of insecticides (pyrethroids, carbamates, organophosphates and organochlorines). The results have demonstrated that *An. funestus* populations were highly resistant to the pyrethroids, deltamethrin and permethrin at all sites tested with mortalities ranging from 0 to 41% and 0 to 44%, respectively in 2015. Concomitant increases in resistance over time to the carbamates bendiocarb (<19%) and propoxur (7%) were also observed. This vector population showed variable response to DDT depending on place and time of testing. This study has also shown that *An. funestus* populations were consistently susceptible (100% mortality) to organophosphates (malathion and pirimiphos-methyl) tested regardless of time and space of testing. Furthermore, this study has shown that *An. arabiensis* was moderately resistant to pyrethroids (57% mortality to deltamethrin in 2015 and <60% mortality to permethrin in the same year) and completely susceptible in some areas.

Results of this study confirm what was earlier reported in a series of cross-sectional studies carried out in the country. The first published report of insecticide resistance in *An. funestus* was on Likoma Island in Lake Malawi in 2011, where this species was found to be resistant to pyrethroids (41.4% mortality to deltamethrin; 40.5% mortality to permethrin) and carbamates (52.5% mortality to bendiocarb; 7% mortality to propoxur) [24]. Similar observations were subsequently reported from the mainland where *An. funestus* populations collected from Chikwawa district in southern Malawi showed resistance to deltamethrin (42% mortality) and permethrin (47% mortality) [19]. Further out in the region, our results compare well with data reported from Tororo in Uganda [25] where resistance to permethrin (62%) and deltamethrin (79%) were also detected. Similar high levels of resistance have been detected in *An. funestus* in Chokwe district in southern Mozambique, where standard exposure time (60 min) to pyrethroids resulted in zero mortality [26]. The finding of *An. funestus* populations being resistant to both pyrethroids and carbamates (bendiocarb and propoxur) in all the study areas is consistent with results reported from several other studies [17, 19, 24, 26–28] except for Uganda [25, 29] where this species was susceptible to carbamates (bendiocarb). Furthermore, the results from Malawi confirm a regional decline in the susceptibility of malaria vectors to pyrethroid and carbamate insecticides [14].

*Anopheles arabiensis*, on the other hand, showed moderate and variable levels of resistance to all insecticides tested. An earlier study carried out in Chikwawa district showed that *An. arabiensis* was completely susceptible to both pyrethroids (permethrin and deltamethrin) [30]. While insecticide resistance appears to be increasing, the overall trends are less striking compared to *An. funestus*. For example, models of *An. funestus* in this study indicate strong increases in resistance to both deltamethrin and permethrin. For *An. arabiensis*, this is only true for permethrin. In western Kenya, *An. arabiensis* consistently had lower levels of resistance to pyrethroid insecticides compared to *An. gambiae* s.s. [31]. Furthermore, this species was less affected by mass distribution of ITNs resulting in a shift in the predominant species in some locations [32]. The lesser impact on *An. arabiensis* compared to *An. gambiae* s.s. and *An. funestus* is presumably due to its less anthropophilic and endophilic tendencies, which allow it to avoid exposure to insecticides applied on nets or the interior walls of houses.

Despite high levels of resistance to pyrethroids and carbamates, *An. funestus* and *An. arabiensis* populations have remained susceptible to organophosphates (malathion and pirimiphos-methyl) in all study sites across the country. Again these results have been reported throughout southern Africa [28] suggesting differential resistance mechanisms relative to pyrethroids and carbamates. It is also likely that organophosphates are less widely used compared to pyrethroid and carbamate insecticides. The response of *An. funestus* and *An. arabiensis* when exposed to DDT was either moderately or completely susceptible likely due to non-use of this insecticide in Malawi following its universal ban due to its well known ecological risks. Earlier results obtained from Chikwawa showed moderate resistance to DDT by *An. arabiensis* populations [30] and it was speculated that there could be selection occurring at larval stage against DDT derivatives that might still be present in the environment.

A reduction over time in susceptibility of *An. funestus* to pyrethroids and carbamate insecticides was observed whether analysed as aggregate level data in a linear regression or using individual mosquito level data in a logistic regression. However, the source of selective pressure is not clear. Logistic regression models indicated no association between the cumulative rounds of IRS and pyrethroid resistance. It is likely that IRS was ceased too early in the monitoring sites to detect an impact. Resistance to pyrethroids was first reported in Malawi in 2011 [24] and in the present study pyrethroid resistance was highest in Nkhotakota district which had received three rounds of IRS with pyrethroids suggesting that excessive use of pyrethroids for IRS had aggravated the resistance to pyrethroids. High ITN coverage the previous year was associated with increased susceptibility to DDT. No other associations were observed between vector control and pyrethroid resistance. However, we note that ITN coverage in Malawi was nil in the year 2000, and has gradually increased over the past 15 years such that in 2015 nearly 70% of the population of the country had access to ITNs.

WHO data also indicate that access to ITNs increased over the course of this study [2]. Changes in the frequency of alleles associated with pyrethroid resistance have been shown to change in response to increasing ITN coverage [31, 33, 34], although the association of phenotypic resistance with increasing ITN coverage has been less well documented. Given enough time, the effect of ITNs on phenotypic resistance may occur even at lower levels of coverage. Lastly, in this study, agricultural use of insecticides was not assessed. In much of rural Malawi, residents are subsistence farmers who do not use much insecticide for crop production although farming of cotton and tobacco does occur and even small scale farmers may use insecticides on these crops. Pyrethroids are commonly used on cotton which is primarily grown in Karonga, Salima, Balaka and Chikwawa districts although this crop is grown throughout Malawi. Inclusion of a variable for cotton-growing districts in the models did not indicate any association with pyrethroid resistance, except for *An. funestus* exposed to bendiocarb, where the odds of mosquito mortality was increased suggesting reduced resistance in cotton growing districts. It was also noted that the level of baseline resistance observed in cotton growing districts was no higher than in areas where cotton agriculture is less prevalent. It has been suggested that inferring agricultural sources of resistance may be done by comparing resistance in larvae versus adults [35]. However, this was beyond the scope of the current study. Despite the uncertainty in the models and given the widespread phenotypic resistance to pyrethroids throughout Malawi, it is likely that ITNs are the primary driver of resistance to these insecticides in Malawi.

Bioassays of *An. funestus* against deltamethrin and permethrin following pre-exposure to PBO indicated higher mortalities in all sites where direct comparisons against bioassays without PBO pre-exposure were done. PBO is an inhibitor of oxidase enzymes that is often used as a synergist in insecticide formulations or as a tool to infer the presence of insecticide resistance mechanisms. The increased susceptibility of *An. funestus* to pyrethroid insecticides suggests that oxidase enzymes are an important mechanism for resistance. These findings are corroborated by molecular studies that have identified several specific oxidase enzymes associated with pyrethroid resistance in *An. funestus* in southern Malawi [36–38]. The restoration of susceptibility to pyrethroids following exposure to PBO suggests that novel long-lasting insecticidal nets (LLINs) such as the PermaNet 3.0 [39] or the Olyset Plus [40] that incorporate this synergist may have improved efficacy in Malawi. Laboratory tests of the PermaNet 3.0 in Mozambique [41] and the Olyset Plus in Malawi [42] confirm that mortality is higher in *An. funestus* exposed to these products compared to similar products without PBO. However, it has yet to be demonstrated that these products are more effective than current generation LLINs under field conditions.

Although carbamates are rarely used in agriculture and, to date, have not been used for public health purposes the observed resistance to this class of insecticides in *An. funestus* was not surprising. It is possible that cross-resistance between the pyrethroids and carbamates is occurring, likely due to elevated activities of metabolic enzymes [43, 44]. Similar resistance patterns to bendiocarb have been observed in *An. gambiae*. The rise of carbamate resistance has precluded the use of bendiocarb for IRS, although use of this insecticide in neighboring Mozambique seemed to be very effective despite high levels of bendiocarb resistance [45].

The development of pyrethroid resistance has important implications, both real and perceived, for malaria control [46]. Pyrethroids have been used for both IRS and LLINs and loss of effectiveness against mosquitoes has serious consequences. For instance, the development of pyrethroid insecticide resistance in *An. funestus* resulted in a sharp increase in malaria cases and consequently necessitated a change to include DDT in combination with pyrethroids for indoor residual spraying (IRS) in South Africa [17]. Although IRS may be implemented with several non-pyrethroid insecticides, most of these are significantly more expensive than pyrethroids. As control programmes have switched to implement non-pyrethroids, the geographic coverage of IRS has decreased and, in some cases, programmes have abandoned IRS altogether [18]. Unfortunately, LLINs are currently only treated with pyrethroids and resistance is a serious concern given that it has been estimated that 68% of the malaria cases averted since 2000 are attributable to LLINs [3]. However, to date, there is limited evidence that pyrethroid resistance is undermining the effectiveness of LLINs [47]. Laboratory experiments in which wild, resistant *Anopheles* strains are directly exposed to net samples have indicated reduced efficacy of LLINs against resistant strains [48–50], while experimental hut studies and field studies have demonstrated that resistant mosquitoes are more likely to enter LLINs, feed upon the occupants and survive [15, 16]. However, to date there is insufficient evidence on the epidemiological significance of the observed pyrethroid resistance on LLINs in the community. A cohort study carried out in an area of moderate pyrethroid resistance in Machinga District in southern Malawi under high net coverage of all sleeping spaces showed that LLINs were effective [51] but a case–control study carried out in the same area among sick children aged <5 years old reporting to the main referral hospital in the area did not find a protective effect of LLINs [52].

This study had several limitations. One potential issue was the use of F_1_ offspring from wild caught mosquitoes for the bioassays which might result in high relatedness among test mosquitoes and a strong correlation in the results. Furthermore, we did not record the number of families included in each set of bioassays. However, an attempt was made to control for family relatedness by accounting for clustering at the bioassay level in the models and calculations of confidence limits. Furthermore, the data span several years and multiple sites which would limit the effect of relatedness in individual bioassays and would suggest the overall results are robust. Similarly, it may be difficult to conclusively determine that the variation observed is due to temporal changes rather than spatial heterogeneity. However, there were a large number of sites sampled that were often close together and models that accounted for spatial heterogeneity indicated there were little or none in the data. Second, the modeling approach to estimate the source of selection may have been inadequate given the uncertainties in insecticide use and the way in which mosquito populations respond to selection. The models may be best suited for rapid changes in resistance whereas low levels of selection may produce similar results over time. The cut-off for high and medium ITN coverage levels was arbitrary and selection may have been more evident if a different cut-off had been selected. Furthermore, the estimates of insecticide use from different sources was likely inaccurate as district level estimates were provided for each factor while fine scale heterogeneity in insecticide use may mask the effect of these different sources of selection. Models which incorporated regional estimates of ITN use gave similar results or inconsistent results with high ITN coverage associated with decreased resistance. Although the models may have been inadequate to clearly pick up the major sources of short-term selective pressure, the widespread increase in pyrethroid resistance would suggest that ITNs are the primary driver of selection as these are the best documented widespread source of pyrethroid insecticides in Malawi.

## Conclusions

*Anopheles funestus* is highly resistant to pyrethroids and carbamates across the country with resistance increasing sharply from 2011 to 2015. *Anopheles arabiensis* was moderately resistant to pyrethroids. There was suspected to moderate resistance of both species to DDT in the country. It is likely that the spread of resistance is driven by the use of ITNs throughout Malawi as these are the only widespread source of insecticide selection pressure in Malawi. New insecticides and new tools are essential to ensure that Malawi can sustain the gains made in malaria control and prevention. It is imperative that resistance monitoring continues in Malawi in order to generate data that would guide deployment of any new malaria control interventions.

## Additional files

**Additional file 1: Table S1.** Approximate locations of villages sampled from 2011 to 2015. Asterisks indicate latitude and longitude were estimated as the average of other villages in the same district or were estimated as the centroid of the district. **Table S2.** Mortality of *An. funestus* in WHO susceptibility tests against deltamethrin. 95% confidence limits and sample size are given in parentheses. **Table S3.** Mortality of *An. funestus* in WHO susceptibility tests against permethrin. 95% confidence limits and sample size are given in parentheses. **Table S4.** Mortality of *An. funestus* in WHO susceptibility tests against bendiocarb. 95% confidence limits and sample size are given in parentheses. **Table S5.** Mortality of *An. funestus* in WHO susceptibility tests against propoxur. 95% confidence limits and sample size are given in parentheses. **Table S6.** Mortality of *An. funestus* in WHO susceptibility tests against DDT. 95% confidence limits and sample size are given in parentheses. **Table S7.** Mortality of *An. funestus* in WHO susceptibility tests against malathion or pirimiphos-methyl (Chikwawa, Ntwana, 2015 only). 95% confidence limits and sample size are given in parentheses. **Table S8.** Mortality of *An. arabiensis* in WHO susceptibility tests against deltamethrin. 95% confidence limits and sample size are given in parentheses. **Table S9.** Mortality of *An. arabiensis* in WHO susceptibility tests against permethrin. 95% confidence limits and sample size are given in parentheses. **Table S10.** Mortality of *An. arabiensis* in WHO susceptibility tests against bendiocarb. 95% confidence limits and sample size are given in parentheses.

**Additional file 2: Figure S1.** Resistance (%) of *An. funestus* populations to Deltamethrin from 2011 to 2015 from various sampling areas across Malawi. Highest levels of phenotypic resistance are denoted by a large open circle and the lowest by the smallest dark dot. **Figure S2.** Resistance (%) of *An. funestus* populations to Permethrin from 2011 to 2015 from various sampling areas across Malawi. Highest levels of phenotypic resistance are denoted by a large open circle and the lowest by the smallest dark dot. **Figure S3.** Resistance (%) of *An. funestus* populations to Bedniocarb from 2011 to 2015 from various sampling areas across Malawi. Highest levels of phenotypic resistance are denoted by a large open circle and the lowest by the smallest dark dot. **Figure S4.** Resistance (%) of *An. arabiensis* populations to Deltamethrin from 2011 to 2015 from various sampling areas across Malawi. Highest levels of phenotypic resistance are denoted by a large open circle and the lowest by the smallest dark dot. **Figure S5.** Resistance (%) of *An. arabiensis* populations to Permethrin from 2011 to 2015 from various sampling areas across Malawi. Highest levels of phenotypic resistance are denoted by a large open circle and the lowest by the smallest dark dot. **Figure S6.** Resistance (%) of *An. arabiensis* populations to Deltamethrin from 2011 to 2015 from various sampling areas across Malawi. Highest levels of phenotypic resistance are denoted by a large open circle and the lowest by the smallest dark dot.
