# Supplementary material for: Increasing insecticide resistance in Anopheles funestus and Anopheles arabiensis in Malawi, 2011–2015
Source: Malar J. 2016 Nov 22;15:563. doi: 10.1186/s12936-016-1610-1 (PMC5120501; doi:10.1186/s12936-016-1610-1)
Supplement: Supplementary file 1 — Additional file 1: Table S1. Approximate locations of villages sampled from 2011 to 2015. Asterisks indicate latitude and longitude were estimated as the average of other villages in the same district or were estimated as the centroid of the district. Table S2. Mortality of An. funestus in WHO susceptibility tests against deltamethrin. 95% confidence limits and sample size are given in parentheses. Table S3. Mortality of An. funestus in WHO susceptibility tests against permethrin. 95% confidence limits and sample size are given in parentheses. Table S4. Mortality of An. funestus in WHO susceptibility tests against bendiocarb. 95% confidence limits and sample size are given in parentheses. Table S5. Mortality of An. funestus in WHO susceptibility tests against propoxur. 95% confidence limits and sample size are given in parentheses. Table S6. Mortality of An. funestus in WHO susceptibility tests against DDT. 95% confidence limits and sample size are given in parentheses. Table S7. Mortality of An. funestus in WHO susceptibility tests against malathion or pirimiphos-methyl (Chikwawa, Ntwana, 2015 only). 95% confidence limits and sample size are given in parentheses. Table S8. Mortality of An. arabiensis in WHO susceptibility tests against deltamethrin. 95% confidence limits and sample size are given in parentheses. Table S9. Mortality of An. arabiensis in WHO susceptibility tests against permethrin. 95% confidence limits and sample size are given in parentheses. Table S10. Mortality of An. arabiensis in WHO susceptibility tests against bendiocarb. 95% confidence limits and sample size are given in parentheses. [file 12936_2016_1610_MOESM1_ESM.docx]

Table S1.

| **District** | **Village** | **latitude** | **longitude** |  |
| --- | --- | --- | --- | --- |
| Chitipa | Unknown | -9.96189 | 33.49934 | * |
| Karonga | Kafikisira | -10.22891 | 34.04450 | * |
| Karonga | Kafwita | -9.80383 | 33.83878 |  |
| Karonga | Ovwa | -10.22891 | 34.04450 | * |
| Karonga | Wovwe | -10.31420 | 34.13807 |  |
| Karonga | Mwenimambwe | -10.35299 | 34.10439 |  |
| Karonga | Fulirwa | -10.44464 | 34.09675 |  |
| Nkhata Bay | Banga | -11.65917 | 34.24250 |  |
| Nkhata Bay | Kande | -12.03768 | 34.05233 | * |
| Nkhata Bay | Matete | -12.03768 | 34.05233 | * |
| Nkhata Bay | Sanga | -12.03768 | 34.05233 | * |
| Nkhata Bay | Mfundi | -12.22056 | 33.98778 |  |
| Nkhata Bay | Dwambazi | -12.23330 | 34.01670 |  |
| Nkhotakota | Chibalepama | -12.31167 | 34.01639 |  |
| Nkhotakota | Matawale | -12.32428 | 34.03864 |  |
| Nkhotakota | Chiputa | -12.34333 | 33.97750 |  |
| Nkhotakota | Vwawa | -12.40932 | 34.09026 |  |
| Nkhotakota | Chapota | -12.47455 | 34.08527 | * |
| Nkhotakota | Mfundi | -12.47455 | 34.08527 | * |
| Nkhotakota | Tawala | -12.47455 | 34.08527 | * |
| Nkhotakota | Chimkwende | -12.98416 | 34.30358 |  |
| Kasungu | Thipa | -12.98455 | 33.38570 | * |
| Mchinji | Mbingwa | -13.69371 | 33.05691 | * |
| Salima | Chikombe | -13.52068 | 34.37870 |  |
| Salima | Mafco | -13.76902 | 34.58713 |  |
| Salima | Mchoka | -13.76936 | 34.60357 |  |
| Salima | Chilungo | -13.80752 | 34.51960 | * |
| Salima | Mang'oma | -13.80752 | 34.51960 | * |
| Salima | Pemba | -13.80752 | 34.51960 | * |
| Salima | Unknown | -13.80752 | 34.51960 | * |
| Salima | Chipoka | -13.91023 | 34.51775 |  |
| Salima | Ngozi | -14.06833 | 34.51083 |  |
| Dedza | Dziko | -14.24891 | 34.60671 |  |
| Dedza | Unknown | -14.27637 | 34.66918 | * |
| Dedza | Chikoleza | -14.28032 | 34.67795 |  |
| Dedza | Mankhande | -14.29990 | 34.72287 |  |
| Mangochi | Chilombo | -14.11644 | 34.92369 |  |
| Mangochi | Nabale | -14.21076 | 35.05439 |  |
| Mangochi | Kela | -14.30133 | 35.11719 |  |
| Mangochi | Mataula | -14.30803 | 35.13897 | * |
| Mangochi | Chipereka | -14.38328 | 35.27895 |  |
| Mangochi | Piyasi | -14.44835 | 35.32062 |  |
| Ntcheu | Chiole | -14.81423 | 34.71307 | * |
| Ntcheu | Dambo | -14.81423 | 34.71307 | * |
| Ntcheu | Nkhwani | -14.81423 | 34.71307 | * |
| Balaka | Chitewere | -14.97620 | 35.22570 |  |
| Balaka | Namwera | -15.00860 | 35.21300 |  |
| Balaka | Chasuchila | -15.08570 | 35.18960 |  |
| Balaka | Sitima | -15.11030 | 35.19260 |  |
| Machinga | Kaudzu | -15.06665 | 35.22543 |  |
| Machinga | Chibwana | -15.07508 | 35.51821 |  |
| Machinga | Sitola | -15.07508 | 35.51821 |  |
| Machinga | Matope | -15.09038 | 35.36358 | * |
| Machinga | Makulisha | -15.14472 | 35.19245 |  |
| Zomba | Kapichi | -15.39859 | 35.42985 | * |
| Phalombe | Mwango | -15.64273 | 35.78565 | * |
| Phalombe | Ndalama | -15.64273 | 35.78565 | * |
| Phalombe | Robert | -15.64273 | 35.78565 | * |
| Phalombe | Sombani | -15.64273 | 35.78565 |  |
| Mwanza | Fumbi | -15.70132 | 34.39927 | * |
| Mwanza | Kayera | -15.70132 | 34.39927 | * |
| Mwanza | Thambani | -15.70132 | 34.39927 |  |
| Chikwawa | Ntwana | -16.01897 | 34.81775 |  |
| Chikwawa | Belo | -16.02083 | 34.81679 |  |
| Chikwawa | Fulirwa | -16.02413 | 34.82353 | * |
| Chikwawa | Kalima | -16.02413 | 34.82353 | * |
| Chikwawa | Kela | -16.02413 | 34.82353 | * |
| Chikwawa | Sisewo | -16.02413 | 34.82353 | * |
| Chikwawa | Medrum | -16.03260 | 34.83606 |  |

Table S2.

| **District** | **Village** | **2011** | **2012** | **2013** | **2014** | **2015** |
| --- | --- | --- | --- | --- | --- | --- |
| Nkhata Bay | Kande | --- | --- | --- | --- | 11 (7-16, 44) |
| Nkhata Bay | Matete | --- | 24 (14-34, 75) | --- | 11 (2-20, 81) | --- |
| Nkhata Bay | Sanga | --- | 21 (13-29, 96) | --- | --- | 14 (6-22, 94) |
| Nkhata Bay | Dwambazi | --- | --- | 12 (0-25, 25) | --- | --- |
| Nkhotakota | Vwawa | 45 (35-55, 100) | --- | --- | 3 (0-6, 32) | 0 (0-22, 15) |
| Nkhotakota | Chimkwende | 50 (39-61, 100) | --- | 19 (15-23, 126) | 2 (0-4, 94) | 8 (3-12, 91) |
| Salima | Chikombe | 75 (33-100, 4) | --- | --- | --- | --- |
| Salima | Mafco | 78 (66-90, 91) | --- | --- | --- | --- |
| Salima | Chilungo | --- | --- | --- | --- | 41 (26-55, 96) |
| Salima | Mang'oma | --- | --- | --- | 21 (12-30, 120) | 8 (0-19, 24) |
| Salima | Chipoka | 52 (33-70, 159) | --- | --- | --- | --- |
| Dedza | Dziko | 54 (41-67, 74) | --- | --- | --- | --- |
| Dedza | Chikoleza | 48 (33-63, 133) | --- | --- | --- | --- |
| Dedza | Mankhande | 65 (46-85, 226) | --- | --- | --- | --- |
| Mangochi | Chilombo | 60 (44-76, 95) | --- | --- | --- | --- |
| Mangochi | Nabale | 72 (53-90, 109) | --- | --- | --- | --- |
| Mangochi | Chipereka | 74 (67-81, 116) | --- | --- | --- | --- |
| Mangochi | Piyasi | --- | --- | --- | --- | 37 (26-48, 122) |
| Balaka | Namwera | --- | --- | 8 (0-19, 25) | --- | --- |
| Balaka | Chasuchila | --- | --- | 18 (1-35, 91) | --- | --- |
| Machinga | Kaudzu | --- | --- | 44 (33-55, 50) | --- | --- |
| Machinga | Chibwana | 41 (24-58, 95) | --- | --- | --- | --- |
| Machinga | Sitola | --- | --- | 31 (23-38, 52) | --- | --- |
| Machinga | Matope | --- | --- | --- | --- | 7 (0-17, 68) |
| Machinga | Makulisha | 42 (31-54, 132) | --- | --- | --- | --- |
| Phalombe | Robert | --- | --- | --- | 11 (0-32, 9) | --- |
| Phalombe | Sombani | --- | --- | 13 (0-29, 16) | --- | --- |
| Mwanza | Thambani | --- | --- | 20 (6-34, 69) | --- | --- |
| Chikwawa | Ntwana | --- | 28 (21-35, 100) | --- | 15 (6-24, 67) | 26 (20-32, 115) |
| Chikwawa | Kalima | --- | --- | --- | --- | 12 (1-23, 99) |
| Chikwawa | Kela | --- | --- | --- | --- | 26 (17-35, 58) |
| Chikwawa | Medrum | --- | 49 (30-69, 99) | --- | --- | --- |

Table S3.

| **District** | **Village** | **2011** | **2012** | **2013** | **2014** | **2015** |
| --- | --- | --- | --- | --- | --- | --- |
| Nkhata Bay | Matete | --- | 93(87-99,100) | --- | 50(28-71,107) | --- |
| Nkhata Bay | Sanga | 94(85-100,32) | 54(28-79,125) | --- | --- | 14 (13-14,37) |
| Nkhotakota | Vwawa | --- | --- | --- | 10(6-14,87) | --- |
| Nkhotakota | Chimkwende | 78(63-94,125) | --- | 46(35-57,100) | 3(0-5,73) | 25 (12-39,103) |
| Salima | Mafco | 84(78-90,50) | --- | --- | --- | --- |
| Salima | Chilungo | --- | --- | --- | --- | 25 (17-34,55) |
| Salima | Mang'oma | --- | --- | --- | 20 (12-29,74) | 32 (18-47,96) |
| Salima | Chipoka | 58 (49-67,100) | --- | --- | --- | --- |
| Dedza | Dziko | 79 (64-93,121) | --- | --- | --- | --- |
| Dedza | Chikoleza | 83 (68-97,103) | --- | --- | --- | --- |
| Dedza | Mankhande | 75 (59-91,48) | --- | --- | --- | --- |
| Mangochi | Nabale | 77 (54-100,13) | --- | --- | --- | --- |
| Mangochi | Chipereka | 77 (68-86,148) | --- | --- | --- | --- |
| Mangochi | Piyasi | --- | --- | --- | 14 (0-28,22) | 44 (42-46,84) |
| Balaka | Namwera | --- | --- | 13 (0-26,24) | --- | --- |
| Balaka | Chasuchila | --- | --- | 67 (54-80,96) | --- | --- |
| Machinga | Kaudzu | --- | --- | 29 (0-58,31) | --- | --- |
| Machinga | Chibwana | 72 (62-81,113) | --- | --- | --- | --- |
| Machinga | Sitola | --- | --- | 23 (18-27,62) | --- | --- |
| Machinga | Makulisha | 68 (50-86,96) | --- | --- | --- | 0 (0-31,10) |
| Phalombe | Robert | --- | --- | --- | 57 (43-71,98) | --- |
| Phalombe | Sombani | --- | --- | 47 (42-52,99) | --- | --- |
| Mwanza | Thambani | --- | --- | 47 (37-57,98) | --- | --- |
| Chikwawa | Ntwana | 80 (74-86,50) | 56 (30-81,126) | --- | 18 (13-24,82) | 29 (20-37,90) |
| Chikwawa | Kalima | --- | --- | --- | --- | 0 (0-31,10) |
| Chikwawa | Medrum | --- | 83 (64-100,75) | --- | --- | --- |

Table S4.

| **District** | **Village** | **2011** | **2012** | **2013** | **2014** | **2015** |
| --- | --- | --- | --- | --- | --- | --- |
| Karonga | Fulirwa | --- | 80 (66-94, 101) | --- | --- | --- |
| Nkhata Bay | Matete | 89 (77-100, 88) | 84 (72-96, 49) | --- | 21 (10-32, 34) | --- |
| Nkhata Bay | Sanga | 78 (53-100, 50) | 80 (71-89, 79) | --- | --- | --- |
| Nkhata Bay | Mfundi | 71 (62-80, 100) | --- | --- | --- | --- |
| Nkhotakota | Chibalepama | --- | 64 (45-83, 25) | --- | --- | --- |
| Nkhotakota | Vwawa | 75 (67-83, 313) | 59 (37-80, 150) | --- | --- | --- |
| Nkhotakota | Mfundi | 84 (70-98, 25) | --- | --- | --- | --- |
| Nkhotakota | Chimkwende | 73 (62-83, 150) | --- | 32 (19-44, 130) | 6 (0-14, 79) | --- |
| Salima | Mchoka | 96 (93-99, 97) | --- | --- | --- | --- |
| Salima | Pemba | --- | 66 (29-100, 50) | --- | --- | --- |
| Salima | Ngozi | --- | 59 (46-73, 101) | --- | --- | --- |
| Dedza | Chikoleza | 41 (27-55, 100) | --- | --- | --- | --- |
| Mwanza | Thambani | --- | --- | 32 (27-38, 74) | --- | --- |
| Chikwawa | Ntwana | 73 (57-89, 70) | 50 (17-83, 92) | --- | 5 (0-11, 75) | 19 (9-29, 100) |
| Chikwawa | Belo | --- | 38 (21-55, 90) | --- | --- | --- |
| Chikwawa | Fulirwa | --- | 96 (88-100, 25) | --- | --- | --- |
| Chikwawa | Medrum | --- | 59 (25-93, 100) | --- | --- | --- |

Table S5.

| **District** | **Village** | **2011** | **2012** | **2013** | **2014** | **2015** |
| --- | --- | --- | --- | --- | --- | --- |
| Nkhata Bay | Matete | 100 (86-100, 25) | 96 (92-100, 75) | --- | --- | --- |
| Nkhata Bay | Sanga | --- | 71 (62-79, 75) | --- | --- | --- |
| Nkhotakota | Chimkwende | --- | --- | 20 (12-28, 75) | --- | --- |
| Chikwawa | Ntwana | --- | 83 (72-94, 100) | --- | 0 (0-4, 91) | 7 (4-10, 57) |
| Chikwawa | Medrum | --- | 88 (81-94, 49) | --- | --- | --- |

Table S6.

| **District** | **Village** | **2011** | **2012** | **2013** | **2014** | **2015** |
| --- | --- | --- | --- | --- | --- | --- |
| Karonga | Fulirwa | 65 (11-100, 75) | --- | --- | --- | --- |
| Nkhata Bay | Matete | 65 (11-100, 75) | 99 (97-100, 93) | --- | --- | --- |
| Nkhata Bay | Sanga | --- | 95 (87-100, 91) | --- | --- | --- |
| Nkhata Bay | Mfundi | 96 (88-100, 25) | --- | --- | --- | --- |
| Nkhotakota | Vwawa | 100 (86-100, 25) | --- | --- | --- | --- |
| Nkhotakota | Chimkwende | 58 (44-72, 50) | --- | 81 (70-92, 100) | --- | --- |
| Salima | Mchoka | 92 (84-100, 75) | --- | --- | --- | --- |
| Salima | Unknown | 84 (70-98, 25) | --- | --- | --- | --- |
| Chikwawa | Ntwana | 89 (81-97, 75) | 94 (91-97, 50) | --- | 100 (92-100, 46) | 75 (71-79, 97) |
| Chikwawa | Belo | --- | 92 (86-98, 50) | 79 (66-92, 63) | --- | --- |
| Chikwawa | Medrum | --- | 100 (86-100, 25) | --- | --- | --- |

Table S7.

| **District** | **Village** | **2011** | **2012** | **2013** | **2014** | **2015** |
| --- | --- | --- | --- | --- | --- | --- |
| Nkhata Bay | Banga | 100 (91-100, 41) | --- | --- | --- | --- |
| Nkhata Bay | Matete | 100 (86-100, 25) | 100 (96-100, 96) | --- | --- | --- |
| Nkhata Bay | Sanga | --- | 100 (95-100, 75) | --- | --- | --- |
| Nkhata Bay | Mfundi | 100 (95-100, 70) | --- | --- | --- | --- |
| Nkhotakota | Matawale | --- | 100 (93-100, 51) | --- | --- | --- |
| Nkhotakota | Chiputa | --- | 100 (86-100, 25) | --- | --- | --- |
| Nkhotakota | Vwawa | --- | 100 (86-100, 25) | --- | --- | --- |
| Nkhotakota | Chapota | --- | 100 (86-100, 25) | --- | --- | --- |
| Nkhotakota | Tawala | --- | 100 (93-100, 51) | --- | --- | --- |
| Nkhotakota | Chimkwende | 100 (95-100, 75) | --- | 100 (86-100, 25) | 100 (95-100, 78) | --- |
| Salima | Mchoka | 100 (86-100, 25) | --- | --- | --- | --- |
| Salima | Ngozi | --- | 100 (86-100, 25) | --- | --- | --- |
| Dedza | Chikoleza | 100 (86-100, 25) | --- | --- | --- | --- |
| Chikwawa | Ntwana | 100 (95-100, 75) | 100 (93-100, 51) | --- | 100 (96-100, 92) | 100 (95-100, 70) |
| Chikwawa | Belo | --- | 100 (93-100, 51) | --- | --- | --- |
| Chikwawa | Medrum | --- | 100 (86-100, 25) | --- | --- | --- |

Table S8.

| **District** | **Village** | **2011** | **2012** | **2013** | **2014** | **2015** |
| --- | --- | --- | --- | --- | --- | --- |
| Chitipa | Unknown | --- | --- | --- | --- | 100 (40-100, 4) |
| Karonga | Kafwita | --- | --- | --- | 58 (31-84, 80) | --- |
| Karonga | Kafikisira | --- | --- | --- | --- | 97 (88-100, 29) |
| Karonga | Ovwa | --- | --- | --- | --- | 95 (85-100, 63) |
| Karonga | Wovwe | 100 (93-100, 50) | --- | --- | --- | --- |
| Karonga | Fulirwa | 100 (86-100, 25) | --- | --- | --- | --- |
| Kasungu | Thipa | --- | --- | --- | --- | 75 (62-88, 100) |
| Mchinji | Mbingwa | --- | --- | --- | --- | 63 (58-67, 40) |
| Salima | Chikombe | 56 (23-88, 9) | --- | --- | --- | --- |
| Salima | Mafco | 75 (50-100, 12) | --- | --- | --- | --- |
| Salima | Mang'oma | --- | --- | --- | 89 (81-97, 107) | --- |
| Dedza | Dziko | 81 (68-94, 73) | --- | --- | --- | --- |
| Dedza | Unknown | --- | --- | --- | --- | 57 (20-94, 7) |
| Dedza | Chikoleza | 67 (40-93, 12) | --- | --- | --- | --- |
| Dedza | Mankhande | 70 (42-98, 10) | --- | --- | --- | --- |
| Mangochi | Chilombo | 100 (54-100, 6) | --- | --- | --- | --- |
| Mangochi | Nabale | 100 (82-100, 19) | --- | --- | --- | --- |
| Mangochi | Kela | 100 (29-100, 3) | --- | --- | --- | --- |
| Mangochi | Mataula | --- | --- | --- | --- | 84 (75-93, 95) |
| Machinga | Kaudzu | --- | --- | 53 (37-68, 93) | --- | --- |
| Machinga | Makulisha | 88 (65-100, 8) | --- | --- | --- | 80 (64-97, 76) |
| Zomba | Kapichi | --- | --- | --- | --- | 89 (81-96, 70) |
| Phalombe | Mwango | --- | --- | --- | --- | 78 (68-88, 96) |
| Phalombe | Ndalama | --- | --- | --- | --- | 65 (60-70, 97) |
| Mwanza | Fumbi | --- | --- | --- | --- | 97 (94-100, 75) |
| Mwanza | Kayera | --- | --- | --- | --- | 97 (93-100, 102) |
| Chikwawa | Sisewo | --- | --- | --- | --- | 69 (61-76, 89) |

Table S9.

| **District** | **Village** | **2011** | **2012** | **2013** | **2014** | **2015** |
| --- | --- | --- | --- | --- | --- | --- |
| Karonga | Kafwita | --- | --- | --- | 40 (20-60, 82) | --- |
| Karonga | Wovwe | 98 (95-100, 50) | --- | --- | --- | --- |
| Karonga | Mwenimambwe | --- | --- | --- | 82 (69-96, 74) | --- |
| Karonga | Fulirwa | 98 (95-100, 50) | --- | --- | --- | --- |
| Nkhata Bay | Matete | 92 (85-99, 115) | --- | --- | 78 (51-100, 9) | --- |
| Nkhotakota | Chimkwende | --- | --- | --- | 48 (14-82, 27) | --- |
| Kasungu | Thipa | --- | --- | --- | --- | 53 (34-71, 68) |
| Salima | Mang'oma | --- | --- | --- | 77 (52-100, 73) | --- |
| Mangochi | Mataula | --- | --- | --- | --- | 58 (45-70, 90) |
| Mangochi | Piyasi | --- | --- | --- | 71 (50-91, 41) | --- |
| Balaka | Chitewere | --- | --- | 91 (79-100, 22) | --- | --- |
| Balaka | Sitima | --- | --- | 83 (67-98, 69) | --- | --- |
| Machinga | Kaudzu | --- | --- | 65 (63-67, 98) | --- | --- |
| Machinga | Sitola | --- | --- | 6 (0-18, 16) | --- | --- |
| Phalombe | Mwango | --- | --- | --- | --- | 47 (40-54, 62) |
| Phalombe | Ndalama | --- | --- | --- | --- | 46 (19-73, 13) |
| Phalombe | Robert | --- | --- | --- | 71 (59-84, 83) | --- |
| Mwanza | Kayera | --- | --- | --- | --- | 100 (93-100, 54) |
| Chikwawa | Ntwana | --- | --- | --- | 56 (42-71, 64) | --- |
| Chikwawa | Sisewo | --- | --- | --- | --- | 44 (12-77, 9) |

Table S10.

| **District** | **Village** | **2011** | **2012** | **2013** | **2014** | **2015** |
| --- | --- | --- | --- | --- | --- | --- |
| Karonga | Kafwita | --- | --- | --- | 56 (42-69, 52) | --- |
| Nkhata Bay | Matete | 100 (92-100, 45) | 100 (95-100, 74) | --- | --- | --- |
| Nkhata Bay | Sanga | --- | 100 (95-100, 74) | --- | --- | --- |
| Salima | Mchoka | 100 (93-100, 50) | --- | --- | --- | --- |
| Machinga | Kaudzu | --- | --- | 99 (97-100, 103) | --- | --- |
| Chikwawa | Medrum | --- | 100 (93-100, 50) | --- | --- | --- |
