# Supplementary figures and images for: Increasing insecticide resistance in Anopheles funestus and Anopheles arabiensis in Malawi, 2011–2015
Source: Malar J. 2016 Nov 22;15:563. doi: 10.1186/s12936-016-1610-1 (PMC5120501; doi:10.1186/s12936-016-1610-1)

## Slide 1
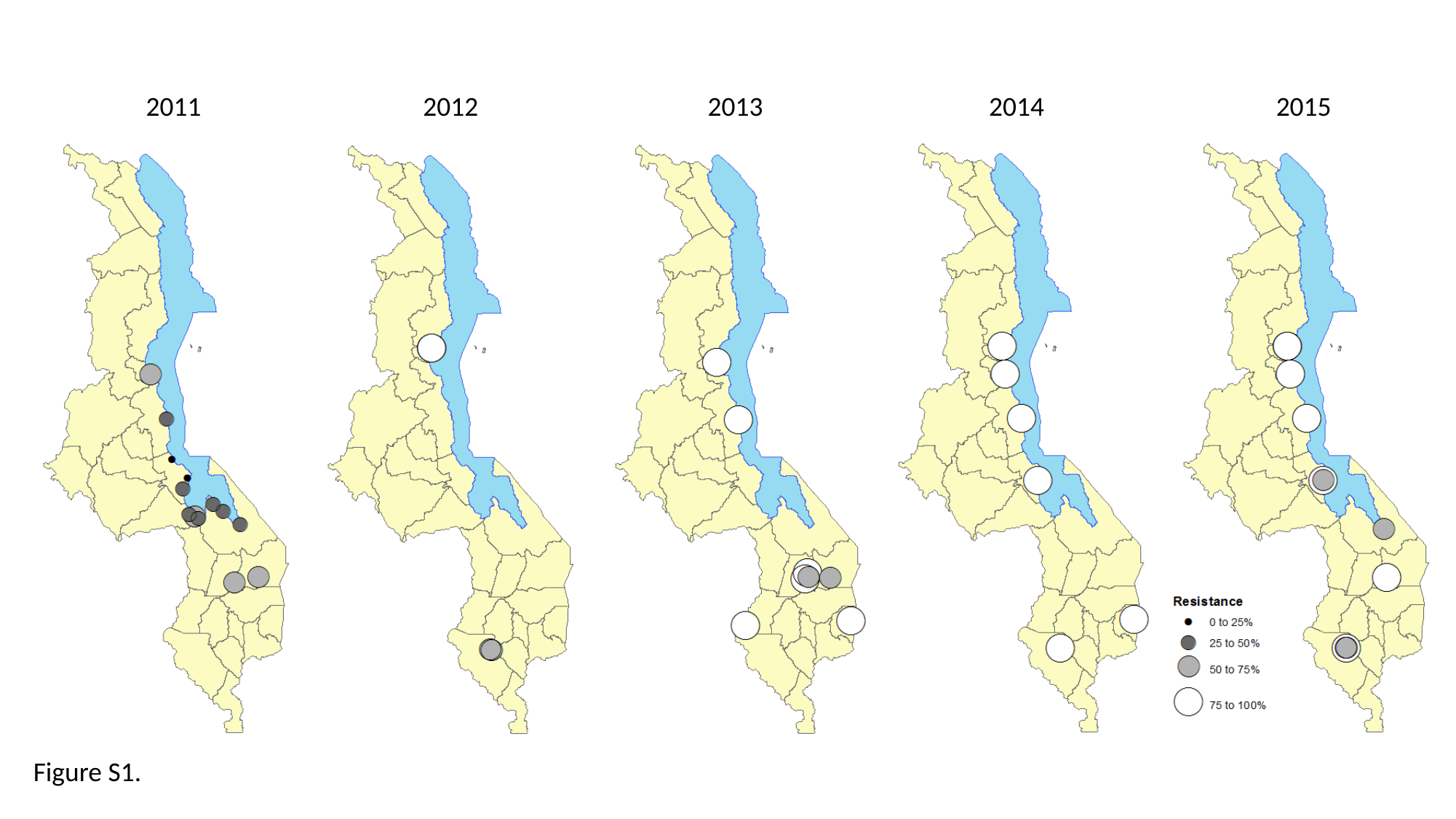

2011
2012
2013
2014
2015
Figure S1.

## Slide 2
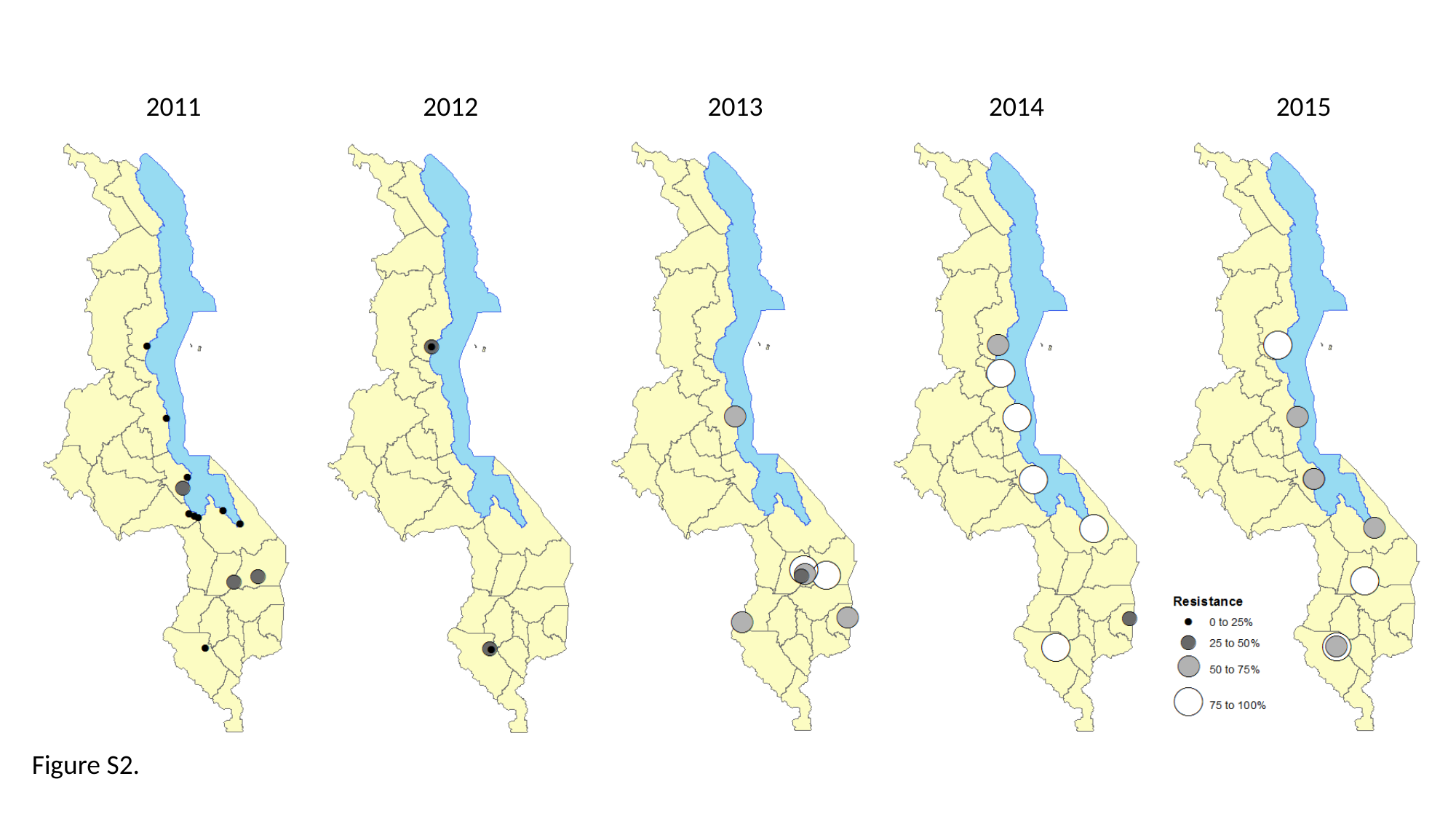

2011
2012
2013
2014
2015
Figure S2.

## Slide 3
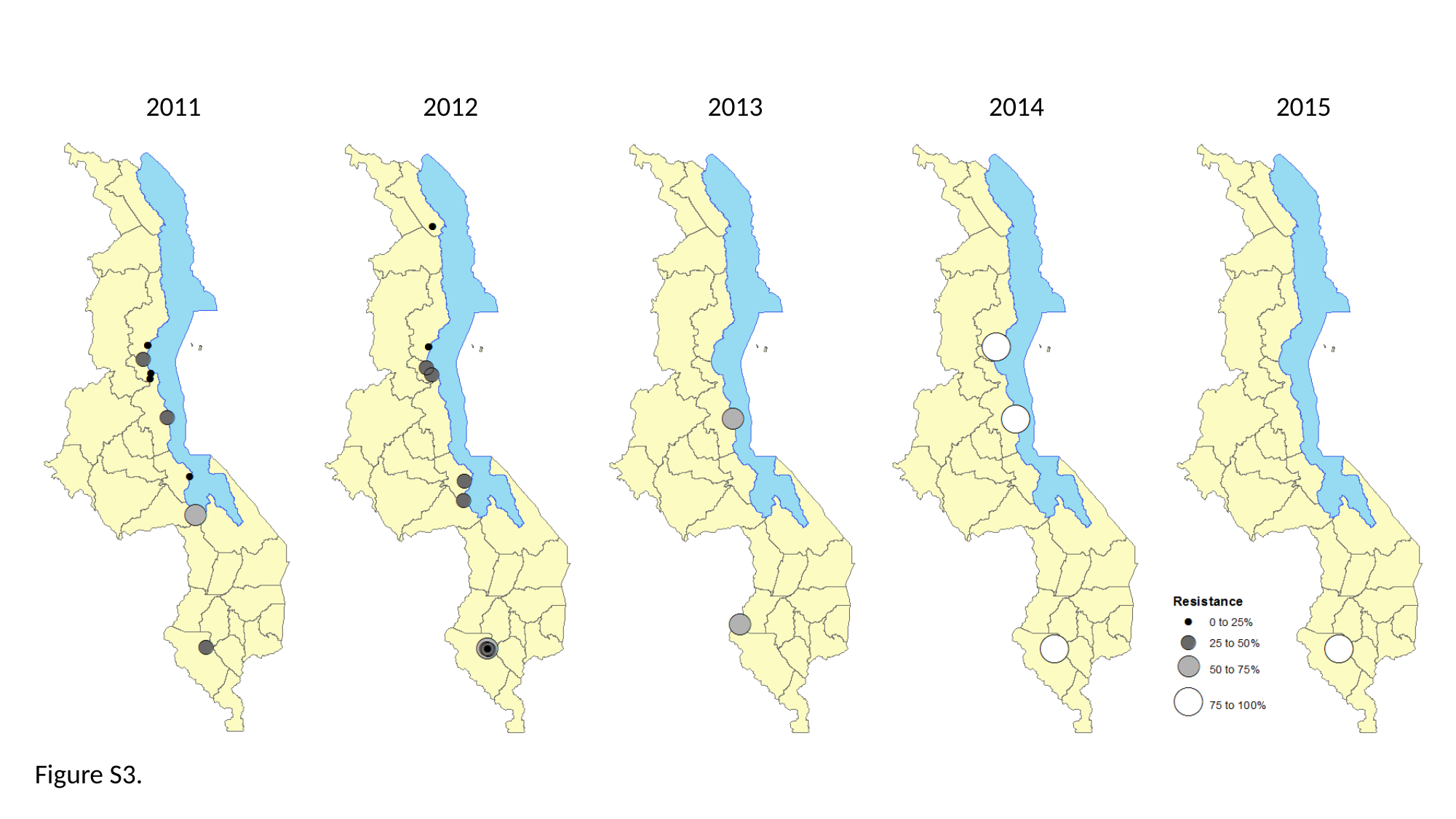

2011
2012
2013
2014
2015
Figure S3.

## Slide 4
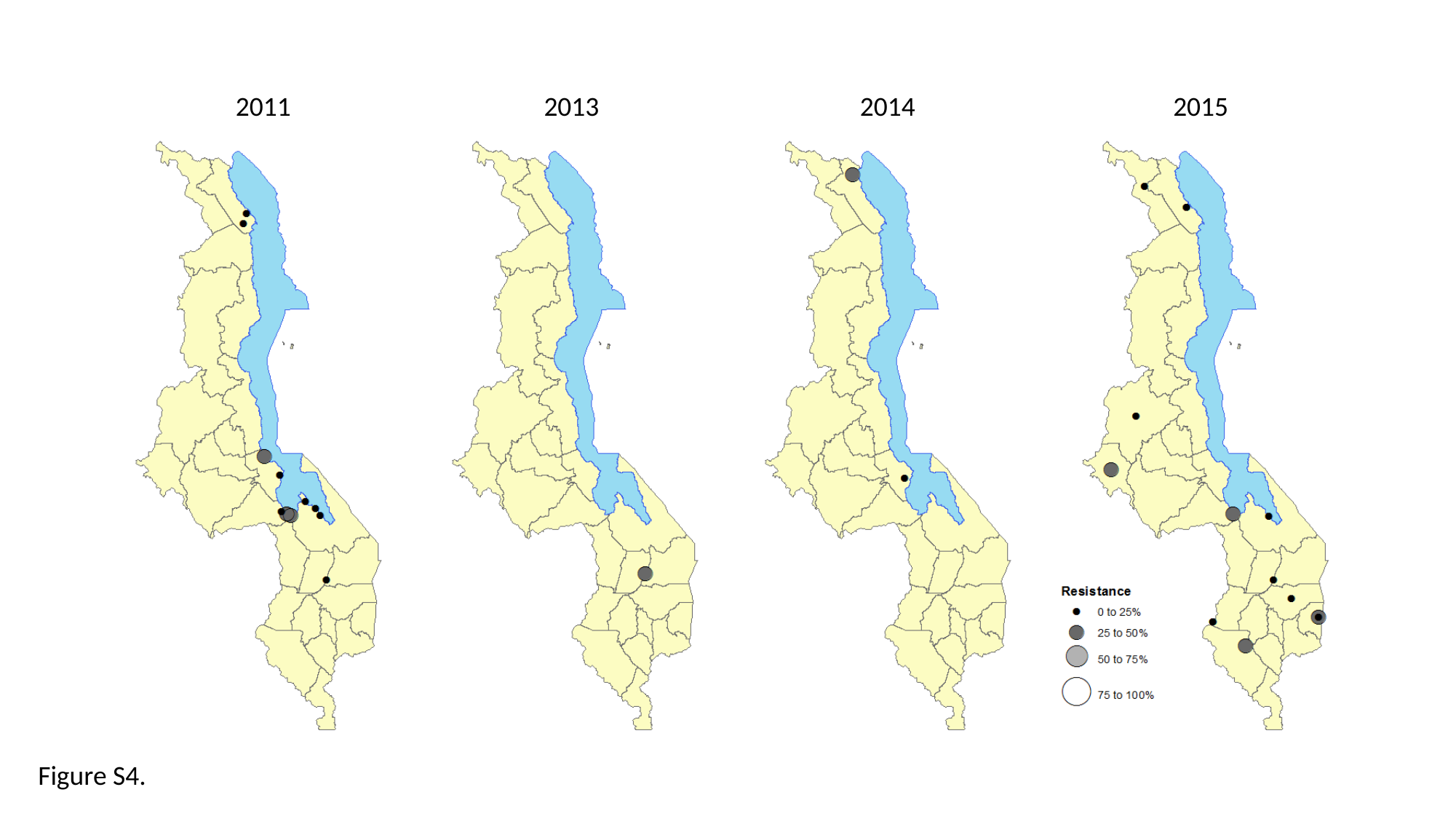

2011
2013
2014
2015
Figure S4.

## Slide 5
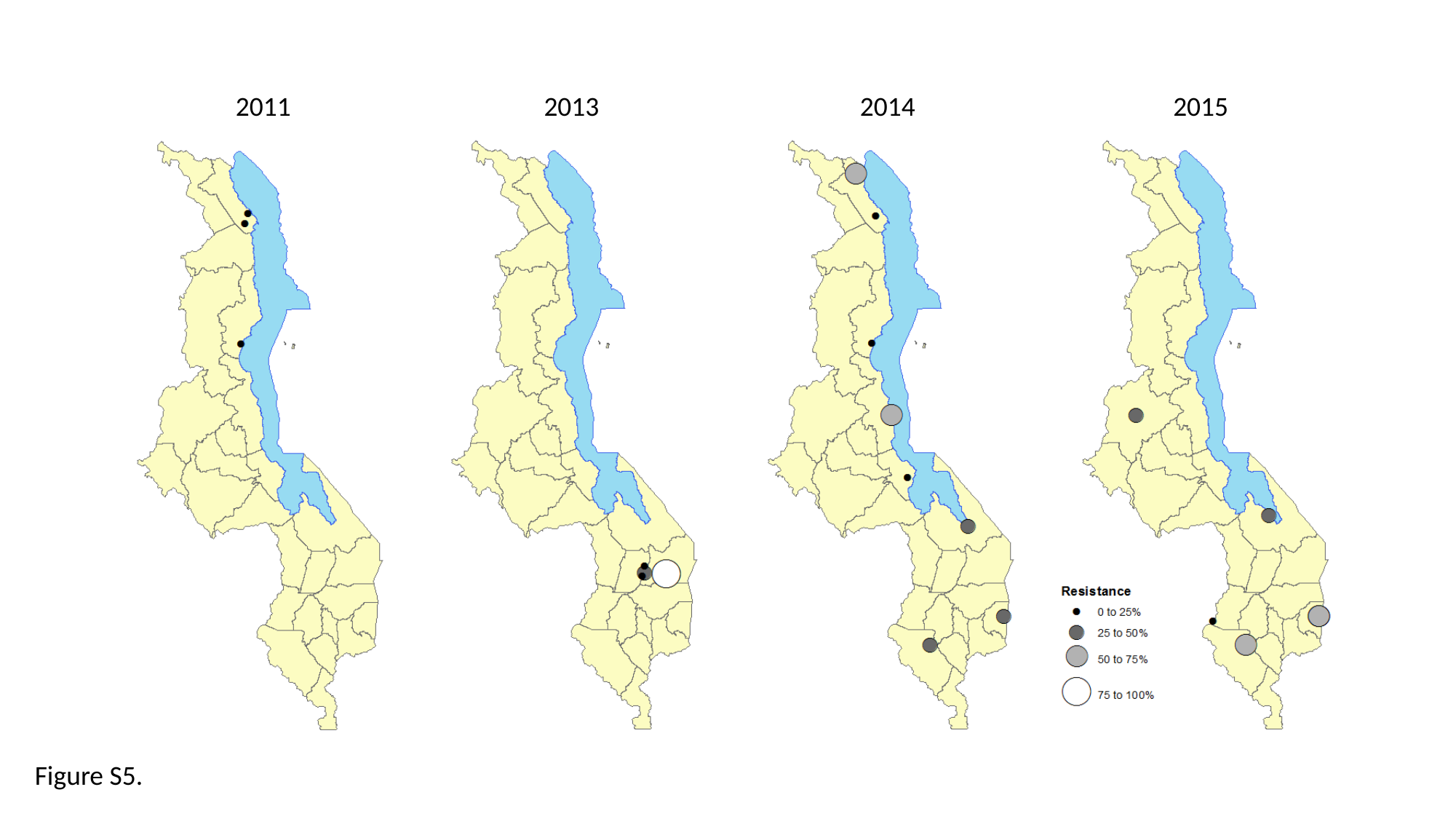

2011
2013
2014
2015
Figure S5.

## Slide 6
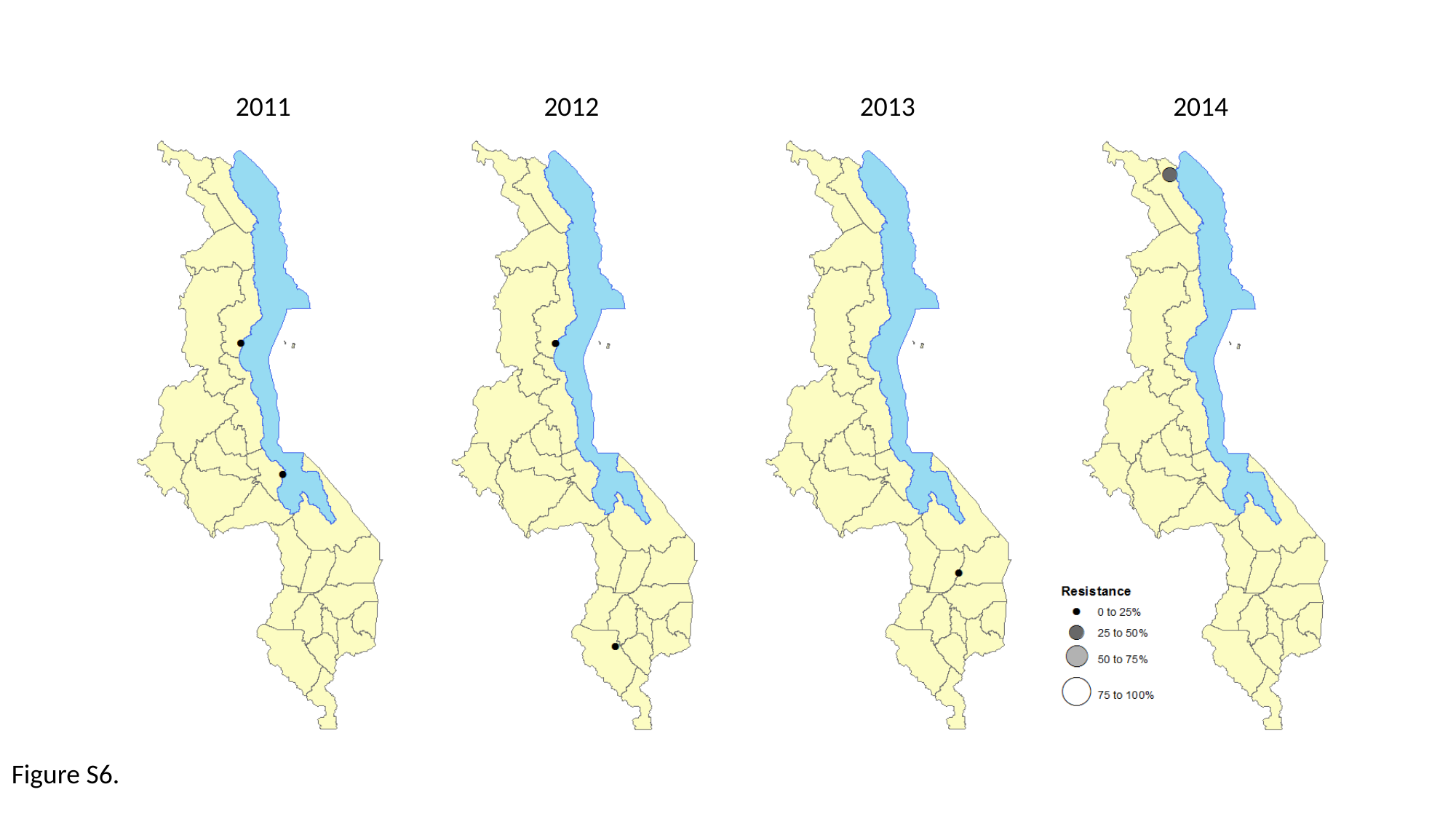

2011
2012
2013
2014
Figure S6.

Supplement: Supplementary file 2 — Additional file 2: Figure S1. Resistance (%) of An. funestus populations to Deltamethrin from 2011 to 2015 from various sampling areas across Malawi. Highest levels of phenotypic resistance are denoted by a large open circle and the lowest by the smallest dark dot. Figure S2. Resistance (%) of An. funestus populations to Permethrin from 2011 to 2015 from various sampling areas across Malawi. Highest levels of phenotypic resistance are denoted by a large open circle and the lowest by the smallest dark dot. Figure S3. Resistance (%) of An. funestus populations to Bedniocarb from 2011 to 2015 from various sampling areas across Malawi. Highest levels of phenotypic resistance are denoted by a large open circle and the lowest by the smallest dark dot. Figure S4. Resistance (%) of An. arabiensis populations to Deltamethrin from 2011 to 2015 from various sampling areas across Malawi. Highest levels of phenotypic resistance are denoted by a large open circle and the lowest by the smallest dark dot. Figure S5. Resistance (%) of An. arabiensis populations to Permethrin from 2011 to 2015 from various sampling areas across Malawi. Highest levels of phenotypic resistance are denoted by a large open circle and the lowest by the smallest dark dot. Figure S6. Resistance (%) of An. arabiensis populations to Deltamethrin from 2011 to 2015 from various sampling areas across Malawi. Highest levels of phenotypic resistance are denoted by a large open circle and the lowest by the smallest dark dot. [file 12936_2016_1610_MOESM2_ESM.pptx]
